# Stereoelectroencephalography Reveals Neural Signatures of Multisensory Integration in the Human Superior Temporal Sulcus during Audiovisual Speech Perception

**DOI:** 10.1523/JNEUROSCI.1037-25.2025

**Published:** 2025-09-10

**Authors:** Yue Zhang, John F. Magnotti, Xiang Zhang, Zhengjia Wang, Yingjia Yu, Kathryn A. Davis, Sameer A. Sheth, H. Isaac Chen, Daniel Yoshor, Michael S. Beauchamp

**Affiliations:** ^1^Department of Neurosurgery, Perelman School of Medicine, University of Pennsylvania, Philadelphia, Pennsylvania 19104; ^2^Department of Neurosurgery, Baylor College of Medicine, Houston, Texas 77030; ^3^Department of Neurology, Perelman School of Medicine, University of Pennsylvania, Philadelphia, Pennsylvania 19104

**Keywords:** audiovisual, multisensory, sEEG, temporal cortex

## Abstract

Human speech perception is multisensory, integrating auditory information from the talker's voice with visual information from the talker's face. BOLD fMRI studies have implicated the superior temporal gyrus (STG) in processing auditory speech and the superior temporal sulcus (STS) in integrating auditory and visual speech, but as an indirect hemodynamic measure, fMRI is limited in its ability to track the rapid neural computations underlying speech perception. Using stereoelectroencephalography (sEEG) electrodes, we directly recorded from the STG and STS in 42 epilepsy patients (25F, 17M). Participants identified single words presented in auditory, visual, and audiovisual formats with and without added auditory noise. Seeing the talker's face provided a strong perceptual benefit, improving perception of noisy speech in every participant. Neurally, a subpopulation of electrodes concentrated in mid-posterior STG and STS responded to both auditory speech (latency 71 ms) and visual speech (109 ms). Significant multisensory enhancement was observed, especially in the upper bank of the STS: compared with auditory-only speech, the response latency for audiovisual speech was 40% faster and the response amplitude was 18% larger. In contrast, STG showed neither faster nor larger multisensory responses. Surprisingly, STS response latencies for audiovisual speech were significantly faster than those in the STG (50 ms vs 64 ms), suggesting a parallel pathway model in which the STG plays the primary role in auditory-only speech perception, while the STS takes the lead in audiovisual speech perception. Together with fMRI, sEEG provides converging evidence that STS plays a key role in multisensory integration.

## Significance Statement

One of the most important functions of the human brain is to communicate with others. During conversation, humans take advantage of visual information from the face of the talker as well as auditory information from the voice of the talker. We directly recorded activity from the brains of epilepsy patients implanted with electrodes in the superior temporal sulcus (STS), a key brain region for speech perception. These recordings showed that hearing the voice and seeing the face of the talker evoked larger and faster neural responses in STS than the talker's voice alone. Multisensory enhancement in the STS may be the neural basis for our ability to better understand noisy speech when we can see the face of the talker.

## Introduction

The process of multisensory integration occurs across modalities and species because it provides a more accurate estimate of the events in the world around us (Stein and Stanford, 2008; Angelaki et al., 2009). A familiar example for humans is speech perception, in which information is conveyed through both the visual modality, in facial movements and gestures, and the auditory modality, in the talker's voice. Multisensory integration is especially beneficial for speech perception under noisy listening conditions, when visual information from the mouth of the talker provides information about speech content that substantially increases perceptual accuracy and decreases listening effort (Sumby and Pollack, 1954; Ross et al., 2007; Peelle and Sommers, 2015).

A framework for the neural basis of multisensory integration was first developed during studies of the role of the superior colliculus in spatial orienting by Stein, Meredith, Wallace and others (Stein and Meredith, 1993; Wallace et al., 1996; Rowland et al., 2007; Stein et al., 2009). These studies documented two neural signatures of multisensory integration. The first is a response to multisensory stimuli that is larger than the response to the component unisensory stimuli presented on their own, especially when the unisensory stimuli are weak or noisy. The second is a faster response for multisensory compared with unisensory stimuli.

While the superior colliculus is critical for multisensory integration during spatial orienting, neuroimaging studies and studies of patients with brain damage have highlighted the superior temporal gyrus (STG) and sulcus (STS) as brain areas that are especially important for speech perception (Dronkers et al., 2004; Poeppel et al., 2008; Price, 2012) and suggested a privileged role for the STS in multisensory integration (Beauchamp et al., 2004; Baum et al., 2012; Zhu and Beauchamp, 2017; Karthik et al., 2024). However, the BOLD fMRI hemodynamic signal is a slow and indirect measure of neural activity. In contrast, invasive recordings from electrodes implanted in the brains of human epilepsy patients (intracranial EEG, iEEG) directly measure neural activity, and the resulting temporal and spatial specificity has greatly increased our understanding of human speech perception (Nourski and Howard, 2015; Yi et al., 2019; Norman-Haignere et al., 2022). Cortex within the STS is inaccessible to the grids and strips of electrocorticographic (ECoG) electrodes that sit on the cortical surface, as used in previous iEEG studies of audiovisual speech (Schepers et al., 2015; Ozker et al., 2017; Karas et al., 2019; Mégevand et al., 2020; Thézé et al., 2020b; Karthik et al., 2024). Recently, clinical practice has transitioned to stereoelectroencephalographic electrodes (sEEG) that pass through an entire brain hemisphere, traversing the STS en route to deep subcortical structures. This change in clinical practice made it possible to use the high spatiotemporal resolution of iEEG to search for the neural signatures of multisensory integration in human STG and STS during audiovisual speech perception.

## Materials and Methods

### Human subjects

All experiments were approved by the Committee for the Protection of Human Subjects at the University of Pennsylvania and Baylor College of Medicine, and participants provided written informed consent. Forty-two participants (25 female, 17 male, mean age 39 years) undergoing Phase 2 epilepsy monitoring were implanted with sEEG electrodes based on clinical criteria for epilepsy localization and resection guidance. All experiments were conducted in the epilepsy monitoring unit, and clinical monitoring continued throughout the experiments.

### Overview

While the theoretical framework for multisensory integration developed in the superior colliculus can be applied to the STG and STS, in collicular recordings, the electrode is advanced until a neuron with the desired properties (responses to both unisensory auditory and visual stimuli) is encountered. Since sEEG electrode shafts are fixed in place, electrodes were selected for analysis that responded to both auditory-only and visual-only speech. The sEEG response in these electrodes was measured in the broadband high-frequency range (70–150 Hz), the signal that best corresponds to the single neuron action potentials measured in colliculus studies (Ray et al., 2008; Dubey and Ray, 2019). Critically, the response to audiovisual speech was not considered in the selection process, allowing for an unbiased comparison between unisensory and multisensory responses (Kriegeskorte et al., 2009).

### Data sharing and reproducibility

The Supplementary Online Material (Data S1) contains an Excel spreadsheet with data and an HTML file with the code to completely reproduce all analyses presented in the manuscript, as well as detailed methods and full results of all statistical tests.

### Word stimuli

**Figure 1.**
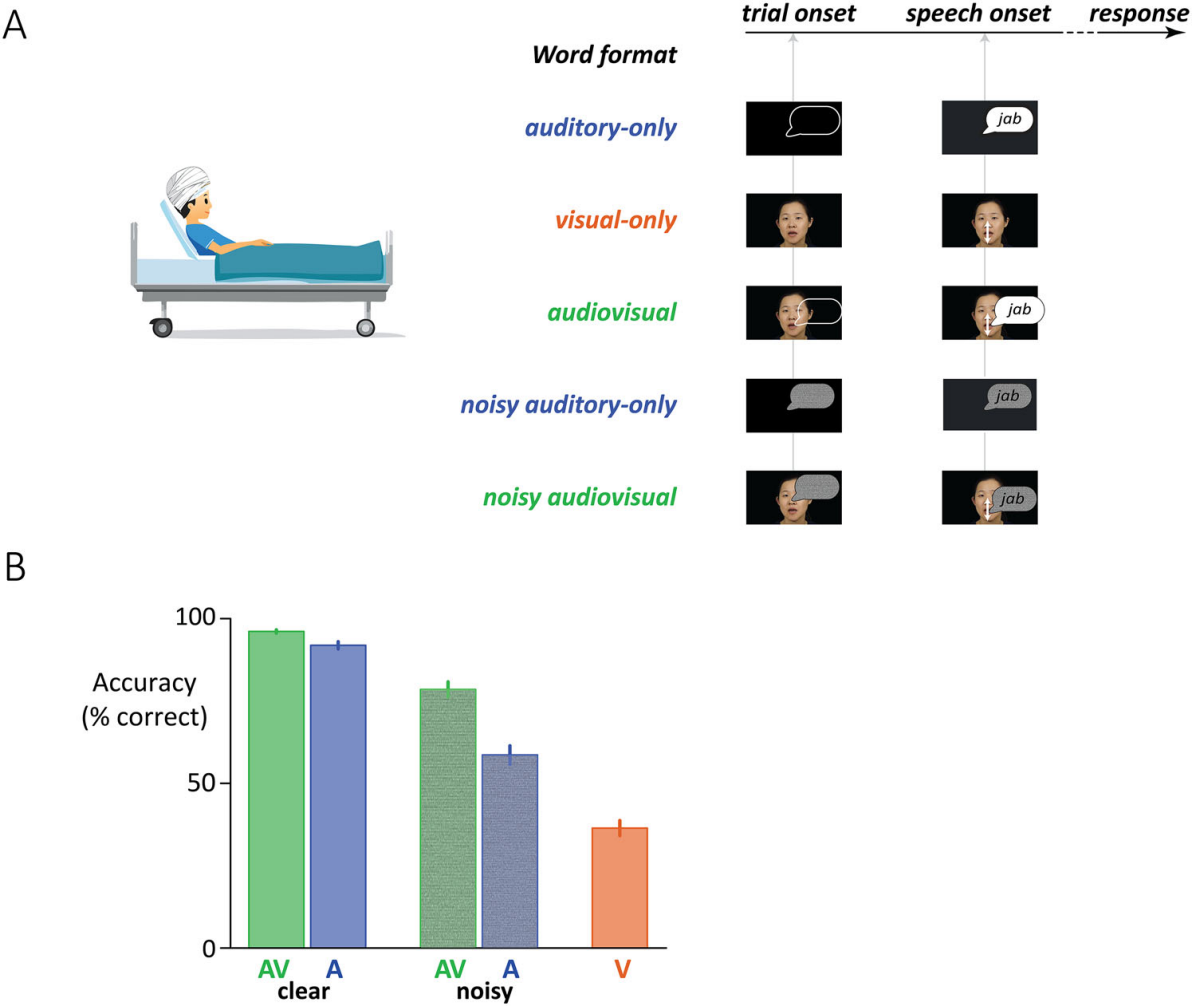
***A***, Participants were presented with single words in one of five formats. Trial onset marked the beginning of stimulus playback. Speech onset occurred at the moment the talker's voice began to pronounce the word (for visual-only words, speech onset was defined as the time when auditory speech began in the corresponding auditory recording). In noisy conditions, auditory pink noise began at trial onset and ended at trial offset (indicated with gray noise pattern inside speech bubble). After word presentation, a blank screen with a white fixation crosshair was presented, signaling participants to repeat back the word. ***B***, Response accuracy for each stimulus format, averaged across all participants (*n* = 42; error bars show standard error of the mean).

Patients rested comfortably in their hospital beds during the experiment. Single words were presented in five formats (Fig. 1): clear auditory speech accompanied by a video of the talker's face (AcV); clear auditory speech accompanied by a white fixation cross in the center of a black screen (Ac); auditory speech with added noise accompanied by a video of the talker's face (AnV); noisy auditory speech accompanied by a white fixation cross in the center of a black screen (An); and a video of the talker's face without any sound (V). Noisy word stimuli were prepared as previously described (Zhang et al., 2023). Videos were presented on an LCD monitor positioned 57 cm from the patient, and auditory speech was played through speakers. Each participant was presented with either 36 or 110 words with noisy speech at an SNR of either −4 dB or −8 dB (see SOM for the details of every word, format, and noise level for every participant).

### Neural recording and preprocessing

sEEG electrodes were implanted using a Robotic Surgical Assistant (ROSA, Zimmer Biomet). Neural signals were acquired at a sampling rate of 2,000 Hz (online bandpass filter of 0.3–500 Hz) using a Blackrock NeuroPort (Blackrock Microsystems) and analyzed using the open source software packages RAVE and YAEL (Magnotti et al., 2020; Wang et al., 2023). Data files containing the raw voltage signals from each electrode were imported into RAVE and preprocessed using the default settings, including notch filters at 60, 120, and 180 Hz applied in the frequency domain to reduce power line interference while minimizing artifacts (Leske and Dalal, 2019), and spectral analysis using Morlet wavelets that varied with frequency to optimize the trade-off between temporal and frequency precision (Cohen, 2014) followed by downsampling to 100 Hz.

### Electrode referencing

Electrodes in or near gray matter were selected for inclusion in the common average reference (CAR) while electrodes outside of the brain or in deep white matter were excluded.

### Trial-based analysis

Data for each trial were epoched to the auditory speech onset for each word, defined as the first positive deflection in the auditory envelope corresponding to the beginning of the speech sound, occurring an average of 742 ± 107 ms (SD) after trial onset (for visual-only trials, the timepoint corresponding to the auditory speech onset in the auditory recording for the same stimulus word was used). For amplitude analysis, the baseline consisted of the 500 ms before that trial's start and the time course of the response in each trial was calculated as the *z*-score of the amplitude (square root of the power) relative to this baseline across the frequency range from 70 to 150 Hz (broadband high-frequency activity, BHA). The time course (in units of *z*-score) was averaged within a window from 0 to 500 ms after the auditory speech onset to obtain a single measurement of BHA for each trial. The response latency was defined as the first time point after speech onset at which the *z*-scored data exceeded *z* = 1.64 (the 95th percentile) of the baseline *z* distribution and remained above this threshold for at least two time points (Raij et al., 2010). The baseline for the latency analysis was the 500 ms window immediately preceding speech onset and was calculated separately for each condition.

### Trial outlier rejection

Outliers were defined at the single trial level, separately for each electrode and blind to the trial type, using the *z*-scored amplitude of the BHA response in the analysis window relative to baseline. The outlier criterion was a response amplitude of 5 median absolute deviations away from the median across all trials. Trials exceeding the outlier criterion were removed, the median was recalculated, and the process was repeated until no more trials met the criterion. Less than 1% of trials were flagged as outliers.

### Anatomical electrode localization and selection

The default workflow of the YAEL software toolbox (Wang et al., 2023) was used to align the preoperative MRI and the postoperative CT. FreeSurfer was used to construct a cortical surface model for each participant (Fischl et al., 1999) and label the location of each electrode with the Desikan–Killiany atlas (Desikan et al., 2006). FreeSurfer uses information about the curvature of the cortical surface to differentiate between gyral (convex, inward curving) and sulcal (concave, outward curving) regions. The total number of electrodes across the 42 participants was 6,739. Electrodes were selected that were within 2 mm of the gray matter of the STG or STS, resulting in a total of 182 electrodes in the STG and 364 electrodes in the STS. Hand labeling was used to assign STS electrodes to either the upper bank (130 electrodes), the fundus (114), or the lower bank (120) of the STS.

### Functional electrode selection

Following anatomical electrode selection, functional criteria were applied. A linear model estimated the auditory-only and visual-only response amplitudes for each electrode. Electrodes that had significant (FDR-corrected *p* < 0.05) responses to both auditory-only and visual-only conditions were classified as “bimodal”; the label “bimodal electrodes” is used as shorthand for “electrodes with measured responses that were significant for both auditory-only and visual-only conditions.” Importantly, the response to audiovisual speech was not used for selection, allowing for an unbiased comparison between unisensory and multisensory responses within bimodal electrodes. Linear mixed-effects models (LMEs) with response amplitude as the dependent variable were constructed to compare conditions. LMEs with response latency as the dependent variable were fit as generalized LMEs (*gamma* family with *log* link) because latencies are always greater than zero (see SOM for complete model specifications).

### Behavioral responses

Participants were instructed to repeat the presented word and responses were manually transcribed. The Jaccard similarity (size of the intersection divided by size of the union) between the phonemes in the presented word and the response was calculated to assign the proportion of correct phonemes (Yu et al., 2024). Responses of “not sure” were scored as 0%. Behavioral data was entered into an LME with fixed effects of word format (auditory-only vs audiovisual) and noise (clear vs added pink noise) with random intercepts for patient and stimulus word. Because accuracy for visual-only words was clearly much lower and they were never paired with pink noise, visual-only accuracy was not included in the LME.

The relationship between iEEG responses and accuracy was assessed in two ways. First, the Pearson’s correlation was calculated across all trials between the participant's accuracy on a given trial and the neural response in that trial (BHA in the window form 0 to 500 ms after auditory speech onset, *z*-scored relative to pretrial baseline, averaged across all bimodal STS and STG electrodes in that participant). Second, an LME was fitted to the same data table with accuracy as the dependent variable; fixed effects of response amplitude, stimulus format (A, V, AV) and auditory noise (with or without added noise) and their interactions; and random intercepts for each participant and a random slope for response amplitude across participants. The *emtrends* function of the *emmeans* package was used for post hoc tests on individual trial types; see SOM for complete analysis code, data table, and statistical output.

## Results

### Perceptual accuracy

Participants were presented with single words in one of five different formats (Fig. 1). Auditory-only trials contained recordings of single words without any visual component, visual-only trials consisted of silent videos of talkers speaking single words, and audiovisual trials combined auditory and visual speech. In the remaining two formats, noisy auditory-only and noisy audiovisual, the auditory speech was masked by pink noise presented for the entire duration of the trial.

On each trial, participants were asked to repeat the presented word, and the phonemes of their response were compared with the phonemes of the presented word to derive an accuracy score. Adding auditory noise reduced perceptual accuracy, from 92% for clear auditory-only speech to 59% for noisy auditory-only speech. Seeing the face of the talker compensated for the effect of auditory noise, increasing accuracy to 78%. The benefit of seeing the talker's face was consistent: for every participant, noisy audiovisual accuracy was higher than noisy auditory-only accuracy. Because accuracy was near ceiling for clear auditory-only speech (92%), seeing the face of the talker provided only limited benefit (96% for clear audiovisual); accuracy was low for visual-only speech (36%).

A linear mixed-effects model (LME) with fixed factors of format (audiovisual vs auditory-only) and auditory noise (clear vs noisy) and random factors of participant and word, found main effects of format (*p* < 10^−16^, driven by greater accuracy for audiovisual speech) and noise (*p* *<* 10^−16^, driven by reduced accuracy for noisy speech). The interaction was also significant (*p* < 10^−16^), driven by the greater benefit of seeing the face of the talker for noisy speech (full results, including parameter estimates, for all LMEs in SOM).

### Identification and characterization of bimodal electrodes

**Figure 2.**
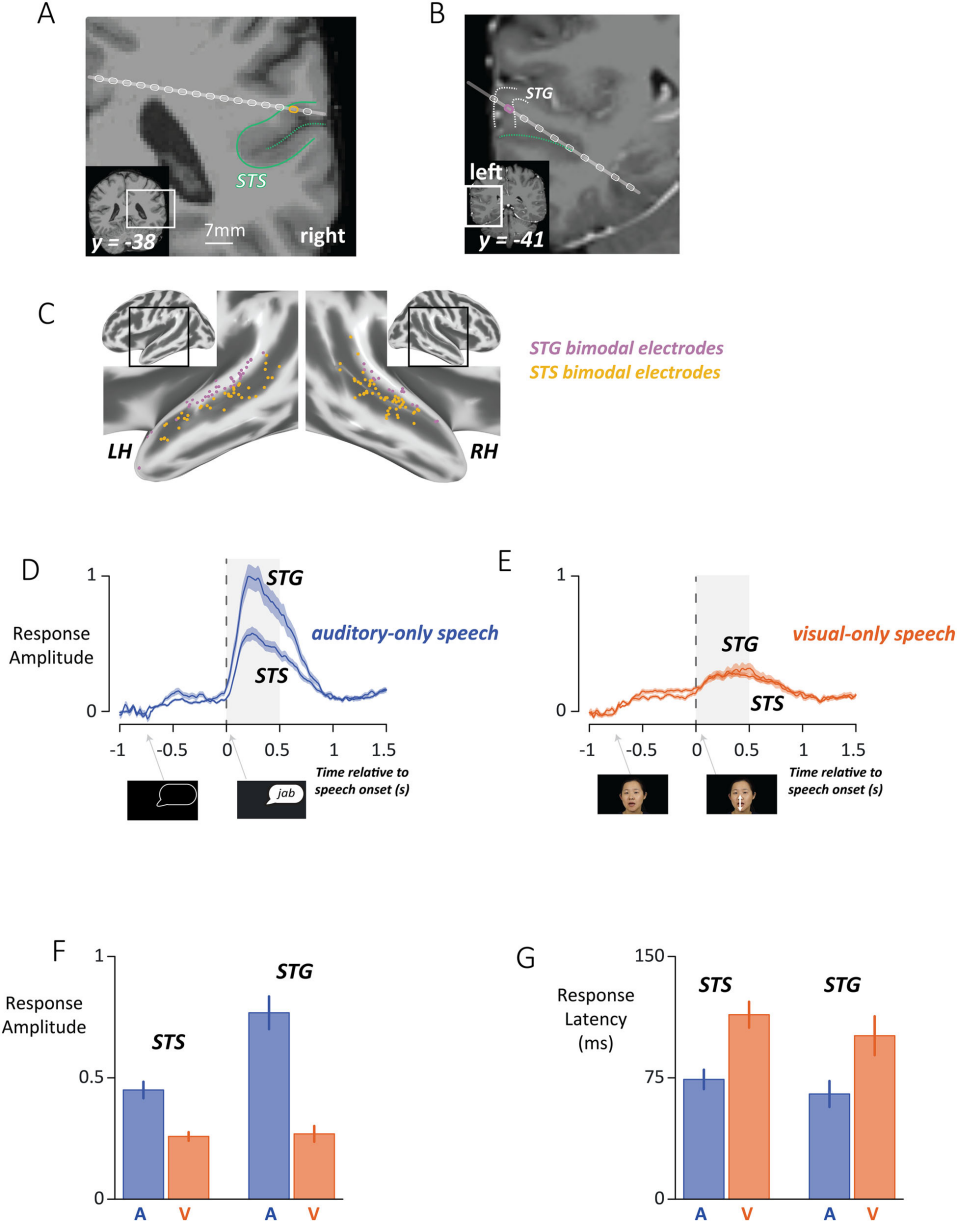
***A***, A coronal slice through a participant's MRI scan with an implanted stereotactic EEG shaft (gray line) containing multiple electrode contacts (white ovals). The superior temporal sulcus (STS) is shown with a green dashed line, the border between cortex in the banks of the STS, and the underlying white matter is shown with a solid green line. One STS electrode (gold oval, electrode code YDY E31) showed a bimodal response pattern, responding both when the patient was presented with auditory-only recordings of words and with visual-only silent videos of words (*p* < 0.05, false discovery rate corrected). ***B***, In a different participant, a stereotactic EEG shaft (gray line) contained a bimodal electrode (purple rectangle, electrode code PAV007 E34) within the superior temporal gyrus (STG, inner and outer borders shown with white dashed lines), superior to the STS (green dashed line). ***C***, Bimodal electrodes were located in both hemispheres in the STS (gold spheres) and the STG (purple spheres), plotted on an inflated cortical surface model of a template brain. ***D***, The response to auditory-only words in the STG (top curve) and the STS (bottom curve), time-locked to speech onset. Dark blue line shows mean; shaded region shows standard error of the mean across electrodes, SEM. Gray shaded region shows analysis window (average *z*-scored power in the frequency band from 70 to 150 Hz from 0 to 0.5 s after speech onset). ***E***, The response to visual-only words (dark orange line, shaded region shows SEM). ***F***, The average response in the analysis window during auditory-only speech (blue bars) and visual-only speech (orange bars) in STS (left) and STG (right). Error bars show SEM. ***G***, The response latency, defined as the first time point at which the response exceeded the baseline, for auditory-only and visual-only speech in STS and STG.

Electrodes in the STS and the STG were identified with significant responses (*p* < 0.05, false discovery rate corrected) to both auditory-only and visual-only speech. Anatomically, bimodal electrodes were located along the length of the upper and lower banks of the STS and STG in both hemispheres (Fig. 2). Out of a total of 364 STS electrodes, 26% were bimodal; 43% responded to auditory-only but not visual-only speech; 6% responded to visual-only but not auditory-only speech; and 25% were inactive. Out of a total of 182 STG electrodes, 29% were bimodal; 49% were auditory-only; 5% were visual-only; and 16% were inactive.

Bimodal electrodes showed increases in broadband high-frequency activity (70–150 Hz) at trial onset, followed by a second, larger response at speech onset, with amplitudes that differed by area and trial type. The speech onset response was quantified using an LME with a dependent measure of average activity in the window from 0 to 0.5 s after speech onset, fixed factors of area (STS vs STG) and trial type (auditory-only vs visual-only). The LME (and all subsequent LMEs on neural responses) used random factors of electrode nested within participant to ensure that the statistical findings could not be dominated by effects in a small number of electrodes or participants. There was a significant main effect of area (*p* = 0.006), driven by stronger responses in the STG, and of format (*p* < 10^−16^), driven by overall larger responses to auditory than visual speech, and a significant interaction (*p* < 10^−16^) driven by similar visual response amplitudes in STG and STS but larger auditory response amplitudes in STG.

Response latency was quantified as the first time after speech onset when the response significantly exceeded the baseline for at least two successive time points. For auditory-only trials, the mean speech onset response latency was 71 ± 5 ms (SEM) while for visual-only trials, the mean speech onset response latency was 109 ± 7 ms. The auditory-only vs visual-only difference was significant (*p* = 10^−5^ for main effect of format in an LME), but STS and STG had similar response latencies (*p* = 0.2 for main effect of area), and there was no interaction between area and format (*p* = 0.9).

### Responses to audiovisual speech in STS bimodal electrodes

**Figure 3.**
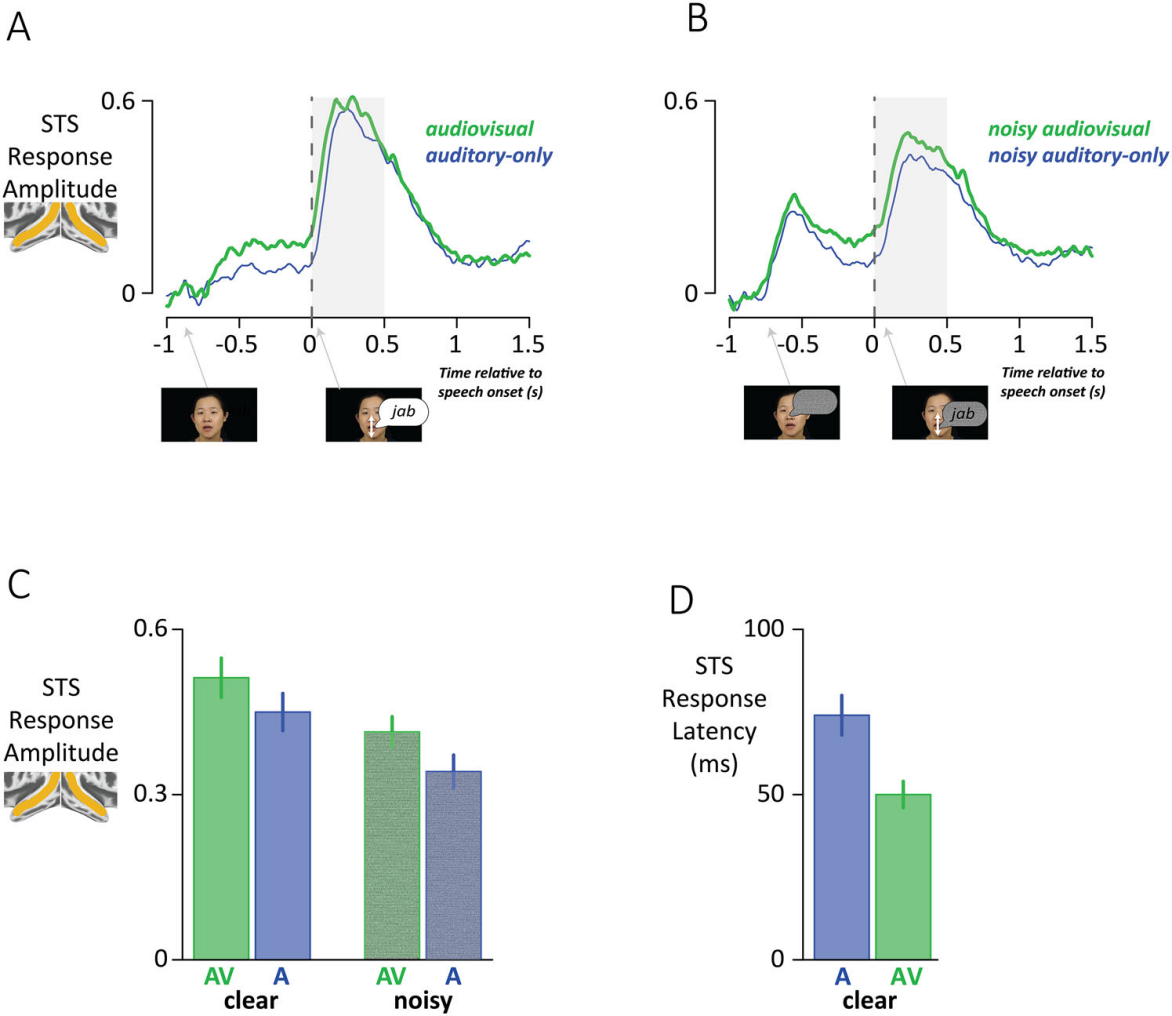
***A***, The time course of the response of STS bimodal electrodes time-locked to speech onset, for audiovisual words (green) and auditory-only words (blue). Thumbnail shows audiovisual stimulus at trial onset and speech onset. Gray shaded region shows analysis window. ***B***, The time course of the STS response to speech with added auditory noise. ***C***, The mean STS response in the analysis window for clear and noisy speech (error bars show SEM across electrodes.) ***D***, The STS response latency for clear auditory-only and audiovisual speech.

STS bimodal electrodes were examined for neural signatures of multisensory integration by comparing the response to audiovisual speech with the response to auditory-only speech (the stronger of the two unisensory responses). As multisensory integration is especially important when sensory signals are weak, responses to both clear speech and speech with added auditory noise were examined (Fig. 3).

In clear speech trials, the speech onset response was larger in audiovisual trials than auditory-only trials. In noisy speech trials, auditory pink noise began at trial onset, evoking a prominent trial onset response, followed by a second response at speech onset that was larger in audiovisual trials than auditory-only trials.

An LME with the dependent variable of the average response in the period from 0 to 0.5 s after speech onset in each condition and fixed factors of auditory noise (clear vs noisy) and format (AV vs A-only) showed a significant main effect of noise (*p* < 10^−16^), driven by weaker responses to noisy speech but no interaction between format and noise (*p* = 0.08). Confirming the theoretical predictions of the multisensory integration framework, there was a significant main effect of format (*p* < 10^−16^) driven by stronger responses to audiovisual than auditory-only speech.

A second indicator of multisensory integration is a shorter response latency (faster response) for multisensory stimuli. Because of the weaker responses to noisy speech, only the latency to clear speech could be calculated reliably. As predicted by multisensory theory, the STS response latency was significantly shorter for audiovisual versus auditory-only speech (50 vs 74 ms, *p* = 10^−15^ for LME, main effect of format).

### Responses to audiovisual speech in STG

**Figure 4.**
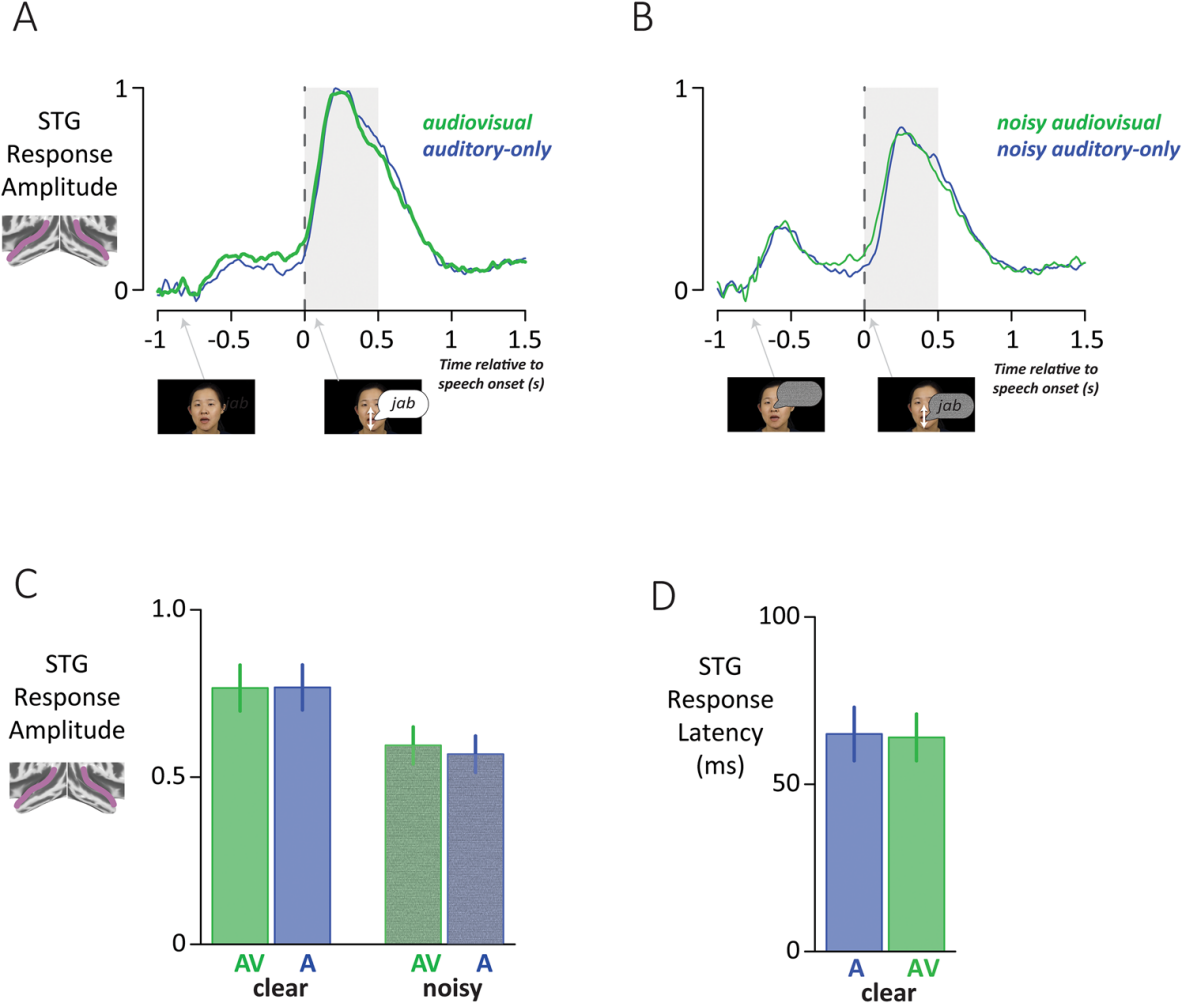
***A***, The time course of the response of STG bimodal electrodes to clear speech. ***B***, The STG response to speech with added noise. ***C***, The mean STG response in the analysis window. ***D***, The STG response latency for clear speech.

The same analyses were repeated for STG bimodal electrodes (Fig. 4). In contrast to the STS, where significant differences between audiovisual and auditory-only speech were observed for both response amplitude and response latency, in the STG no significant differences were observed (*p* = 0.19 for main effect of format from the LME on amplitude and *p* = 0.76 for the main effect of format from the LME on latency; see SOM for full LME results). The STG response latency was 64 ms in the audiovisual condition and 65 ms in the auditory-only condition.

### Comparison of multisensory effects in STS and STG

In the STS, audiovisual speech evoked significantly larger and faster responses than auditory-only speech, while in the STG there were no significant differences between formats. However, because the difference between “significant” and “not significant” is not necessarily significant (Gelman and Stern, 2006), additional analyses compared the areas directly.

An LME for response amplitude was constructed with main effects of area (STS vs STG), condition (audiovisual vs auditory-only), noise, and all interactions. There were significant interactions between area and condition and between area and noise. Post hoc tests confirmed that the audiovisual versus auditory-only amplitude difference was significantly greater in the STS than STG for both clear speech (*p* = 10^−4^) and noisy speech (*p* = 0.003).

**Figure 5.**
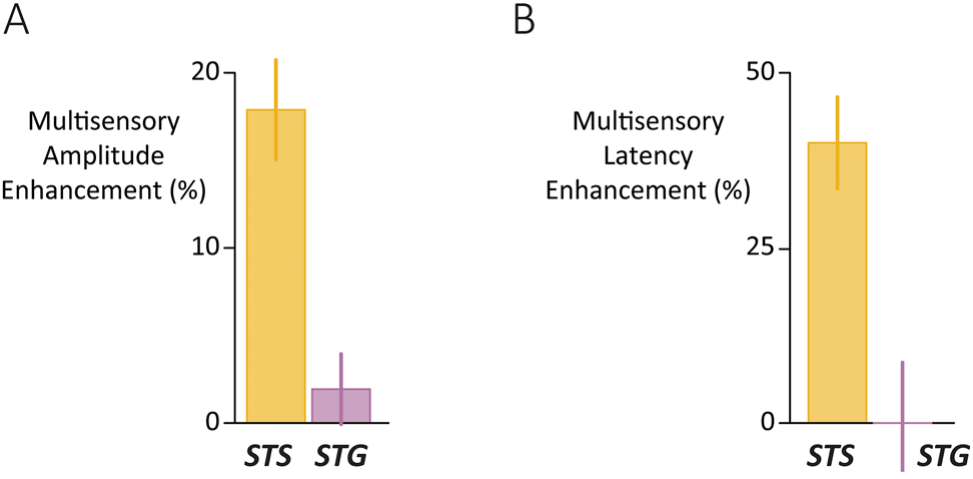
***A***, For each electrode, the auditory-only response amplitude was used as the baseline, and the relative increase for audiovisual speech was calculated using the formula 100 * ((AV − A)/A). ***B***, For each electrode, the relative audiovisual versus auditory-only response latency was calculated using the formula 100 * ((A − AV)/A). Height of bar corresponds to latency decrease for audiovisual compared with auditory-only speech.

A multisensory amplitude enhancement index was calculated for each electrode, defined as the percent change between the audiovisual response and the auditory-only response, averaged across clear and noisy speech (Fig. 5). The index was higher for STS electrodes than STG electrodes: 18% versus 2%.

An LME for response latency also found a significant interaction between area and format (*p* = 0.007) driven by significantly faster responses for audiovisual compared with auditory-only speech in the STS (50 vs 74 ms, *p* = 10^−5^) but not the STG (64 vs 65 ms, *p* = 0.8). The multisensory latency enhancement index (defined as the percent change between the auditory-only latency and the audiovisual latency) was calculated for each electrode. The median STS index was 40%, meaning that the audiovisual response was 40% faster than the auditory-only response, but the median STG index was 0%.

Bimodal electrodes in the STS showed both multisensory amplitude increases and latency decreases. Correlating the indices for each effect across electrodes revealed a weak relationship (*r* = 0.04, *p* = 0.69), suggesting that the effects were largely independent.

### Anatomical effects: STS subregions

STS was further subdivided into three subregions consisting of the upper bank of the sulcus (*n* = 130), the fundus of the sulus (*n* = 114), and the lower bank of the sulcus (*n* = 120), and the main analyses were repeated.

A *X*^2^ test of independence comparing the relative frequency of electrode type for each subregion yielded a significant effect ( $X_{6}^{2}$ = 40.5, *p* = 10^−7^). A generalized LME (binomial family) revealed a higher likelihood for bimodal electrodes in the upper bank compared with the fundus (*p* = 0.004) or the lower bank (*p* = 10^−4^), but no difference between fundus and lower bank (*p* = 0.3).

For multisensory response enhancement, an LME with fixed effects of format (A vs AV), noise (clear vs auditory noise), and subregion (upper bank, fundus, lower bank) yielded no format-by-subregion interaction (*p* = 0.5), nor three-way interaction (*p* = 0.7), suggesting similar enhancement effects. There was a main effect of subregion (*p* = 0.002), driven by overall larger responses in the upper bank versus fundus (*p* *=* 0.001), and a significant interaction between noise and subregion (*p* = 10^−5^), driven by greater reduction in response for noisy response in the upper bank compared with the lower bank (*p* = 10^−4^) and the fundus (*p* = 10^−5^). The main effects of format (*p* < 10^−16^) and noise (*p* < 10^−16^) were the same as those observed in the analysis of the entire STS described above.

For multisensory latency enhancement, the LME yielded main effects of format (*p* = 10^−5^) and subregion (*p* = 0.005; upper bank = 55 ± 4 ms, lower bank = 60 ± 9 ms, fundus = 79 ± 9 ms). Critically, there was no interaction between format and subregion (*p* = 0.62), suggesting similar latency enhancement across subregions.

### Anterior-to-posterior differences

Bimodal electrodes were broadly distributed but concentrated in the middle and posterior regions of the STS and STG (Fig. 2*C*). An LME with the dependent measure of standard space *y* coordinate, and fixed effects of electrode type (bimodal electrodes vs all other electrodes, including auditory-only, visual-only, and inactive electrodes) and area (STS vs STG) revealed a significant effect of electrode type (*p* = 10^−6^) driven by a more posterior average location for bimodal electrodes (mean *y* = −28 mm) compared with other electrodes (*y* = −24 mm). The STS extends more posteriorly than the STG, resulting in a main effect of area (*p* = 10^−6^), but no significant interaction between electrode type and area (*p* = 0.6).

This analysis demonstrated that bimodal electrodes are located more posteriorly than other electrode types, raising the question of an anatomical gradient within the population of bimodal electrodes. Data were combined across STS subregions and STG to increase statistical power (the total of 146 bimodal contacts provided 80% power to detect correlations of at least 0.23). For unisensory response amplitudes, there was a correlation between stronger visual-only responses and more posterior locations (*r* = 0.45, *p* = 10^−8^) but not for auditory-only responses (*r* = 0.07, *p* = 0.4).

A regression on audiovisual enhancement (AV–A) in each electrode showed a significant relationship with the strength of the visual-only response in that electrode (*p* = 10^−10^) but not the anterior-to-posterior location (*p* = 0.6).

For response latency, no significant associations were found between anterior-to-posterior location and response latency for unisensory auditory speech, audiovisual speech, or the audiovisual speedup.

### Brain–behavior relationship

To assess the relationship between brain activity and behavior, we examined the phonetic accuracy of the perceptual report of the participant for each single trial (measured as percent correct) and compared it with the neural amplitude of response (measured as the broadband response in the speech onset analysis window, averaged across all STS and STG bimodal electrodes in that participant for that trial). Simply correlating these two values produced a result with high statistical significance (*r* = 0.15, *p* < 10^−16^). However, this correlation was confounded by differences between trial types—audiovisual trials had high accuracy and large neural responses, while the opposite was true for visual-only trials. A second analysis consisted of an LME with dependent variable of the phonetic accuracy of the response in each trial; fixed factors of trial type, auditory noise, amplitude of the neural response in each trial, and their interactions; and random intercepts for each participant. There were significant main effects of trial type (*p* < 10^−16^) and noise (*p* < 10^−16^) and the trial type by noise interaction (*p* < 10^−16^). Although there was no main effect of neural response amplitude, there was a three-way interaction between trial type, auditory noise, and amplitude. Examining the relationship between response amplitude and accuracy separately for each trial type revealed larger amplitude responses correlated with higher accuracy for auditory-only noisy trials (*p* = 0.007) and visual-only trials (*p* = 0.03), but not for the other trial types (all *p*s > 0.18). This may be due to the fact that noisy auditory and visual-only trials were the only trial types with substantial numbers of errors and hence adequate statistical power to detect the correlation between response amplitude and accuracy.

## Discussion

Recordings from single neurons in the superior colliculus during spatial orienting established a theoretical framework for multisensory integration (Stein and Meredith, 1993). In sEEG recordings from the human STS and STG during speech perception, bimodal electrodes in the STS displayed the signatures of multisensory integration predicted by the framework, including significantly faster and larger responses to audiovisual speech compared with auditory-only speech. In contrast, STG bimodal electrodes showed neither faster multisensory latencies nor significant response enhancement.

### Mechanisms for the multisensory latency advantage

Within STS electrodes, the response latency for audiovisual speech was significantly faster than the response latency for auditory-only speech (50 ms vs 74 ms). However, the response latency for visual-only speech was 109 ms. How could visual speech information influence auditory speech processing if it arrives later? The answer is that visual information was available starting at trial onset, an average of 742 ms before speech onset; the neural response to trial onset is clearly visible in the visual-only and audiovisual time courses. For many phonemes, talkers make preparatory mouth movements tens or hundreds of milliseconds before speech onset (Chandrasekaran et al., 2009). This “visual head start” allows the auditory system to prepare for the incoming auditory speech and can provide information in advance about speech content, because a given preparatory mouth shape is most compatible with a limited set of phonemes (Karas et al., 2019).

### Comparison with previous electrophysiological studies of audiovisual speech perception

Published iEEG studies of audiovisual speech perception sampled activity in the STG using surface grids of ECoG electrodes (as opposed to the penetrating sEEG electrodes used in the present study). Karas and colleagues presented four stimulus words and found that STG audiovisual response amplitudes measured with ECoG were significantly weaker than auditory-only responses for two of the stimulus words and similar for the other two stimulus words (Karas et al., 2019). Metzger and colleagues presented audiovisual speech in which the asynchrony between auditory and visual speech was varied, finding that STG responses were best fit by a model with weaker responses to audiovisual than auditory-only speech (Metzger et al., 2020). Karthik and colleagues presented 12 different phonemes and found no significant differences in high gamma power between auditory-only and audiovisual conditions following speech onset in either anterior STG, middle STG, or posterior STG (Karthik et al., 2021). In summary, there is evidence for major differences in multisensory effects between the STG and the STS. Published studies are consistent with the absence of multisensory enhancement in the STG noted in the present study (with two studies finding evidence for multisensory suppression in STG), while the STS showed significant multisensory enhancement.

One published study used sEEG and ECoG to record responses to auditory-only speech (but not audiovisual speech) in the STS and STG (Nourski et al., 2021). As in the present study, response latencies to auditory-only speech were slower, and response amplitudes weaker, in the STS than in the STG.

The present study analyzed the iEEG broadband high-frequency signal (70–150 Hz) as the best proxy for single neuron action potentials. The power and phase in lower-frequency bands has been implicated in multisensory integration during speech perception, especially entrainment to syllable-rate components (Senkowski et al., 2008; Giraud and Poeppel, 2012; Peelle and Davis, 2012; Hyafil et al., 2015; Ohki et al., 2016; Thézé et al., 2020b; Karthik et al., 2021; van Bree et al., 2021; Aller et al., 2022; Michail et al., 2022).

While our approach examined iEEG responses at individual electrodes in the STS and STG, in EEG a valuable approach is to examine data from whole-head 128-channel recordings to demonstrate multisensory interactions using regularized regression (Di Liberto et al., 2018; O’Sullivan et al., 2021). An important finding from these studies is that multisensory integration during speech perception occurs over long temporal windows of hundreds of milliseconds (Crosse et al., 2016). This is consistent with the time courses observed in the present study, in which the divergence between the auditory-only and audiovisual responses endured for more than a second, from soon after trial onset until after speech offset (Fig. 3).

In a scalp EEG examination of the N1 and P2 event-related potentials (van Wassenhove et al., 2005), compared audiovisual and auditory-only speech noted both latency decreases of ∼20 ms and amplitude decreases spanning ∼200 ms, and an MEG study reported a similar effects (Arnal et al., 2009).

### Functional heterogeneity in STS and STG

Bimodal electrodes were broadly distributed throughout the anterior to posterior extent of STS and STG, with a concentration in mid to posterior regions. This supports the idea that responses to visual speech and multisensory interactions are more common in posterior lateral temporal cortex, as observed in previous iEEG studies (Ozker et al., 2017, 2018; Karas et al., 2019; Metzger et al., 2020; Karthik et al., 2021). Seeing the talker’s mouth open provides a useful cue about the timing of auditory speech onset, consistent with the finding that posterior regions of the STG respond preferentially to auditory speech onset (Hamilton et al., 2018).

Bimodal sEEG electrodes were found intermingled with electrodes that responded only to auditory-only speech, only to visual-only speech, or to neither. This is consistent with a high-resolution BOLD fMRI study (Beauchamp et al., 2004) describing a patchy organization within human STS and STG, with individual patches responding to auditory-only speech, visual-only speech, or audiovisual speech. The responses of these voxels were consistent across different imaging runs and different stimulus material, demonstrating that the selectivity was not a statistical artifact. A 7 T BOLD fMRI study also noted a patchy organization of visual responses in auditory cortex (Gau et al., 2020). In a single-unit recording study in nonhuman primates (NHPs; Dahl et al., 2009), individual STS neurons showed bimodal, auditory-only, or visual-only response patterns, and neurons with similar preferences were clustered together on the scale of millimeter, as required to produce a patchy organization measurable with techniques that record aggregate neural activity, such as BOLD fMRI and sEEG. In single NHP STS neurons, the most common multisensory interaction was a subadditive multisensory enhancement (meaning the audiovisual response was less than the sum of the auditory and visual responses) as observed for the STS the present study.

### Relationship to perception

A multivariate fMRI analysis applied across broad swaths of STS and STG decoded the stimulus above chance for both auditory and visual speech (Karthik et al., 2024). Applying a multivariate analysis to the entire STS and STG, Zhang and colleagues showed that intelligible noisy speech evoked a BOLD fMRI activation pattern similar to that of clear speech (Rennig and Beauchamp, 2022; Zhang et al., 2023). Transcranial magnetic stimulation (TMS) studies also support a causal role for the STS in audiovisual speech perception (Beauchamp et al., 2010; Ahn et al., 2024).

Consistent with decades of behavioral speech research, seeing the face of the talker improved the accuracy of speech perception, with every participant showing greater accuracy for noisy audiovisual than noisy auditory-only speech (Sumby and Pollack, 1954; Peelle and Sommers, 2015). However, much less is known about the speed of speech perception. The neural recordings showed significantly shorter latency responses for audiovisual speech (fastest latency of 50 ms in the STS) compared with auditory-only speech (fastest latency of 65 ms in the STG). The latency advantage for audiovisual speech raises the interesting possibility that the maximum rate at which speech can be perceived is higher for audiovisual than auditory-only speech, presupposing a model in which STS/STG processing speeds are a rate-limiting step in speech perception. Audiovisual speech of varying speeds could be created by asking talkers to voluntarily adjust their talking speed, by digitally manipulating the talking speech of real talkers, or by adjusting the talking speed of synthetic avatar faces (Thézé et al., 2020a; Varano et al., 2021; Shan et al., 2022; Yu et al., 2024).

### Implications for neuroanatomical models of speech perception

For auditory-only speech, STG latencies were shorter than STS latencies (65 ms vs 74 ms) while for audiovisual speech, STS latencies were shorter than STG latencies (STS: 50 ms vs STG: 64 ms). This difference raises the possibility of a parallel processing model in which the STG plays the primary role in auditory-only speech perception, while the STS takes the lead in audiovisual speech perception (Rauschecker, 1998; Peelle et al., 2010; Davis and Johnsrude, 2003; Hickok and Poeppel, 2004; Hamilton et al., 2021). This model could be tested using electrical brain stimulation delivered through sEEG electrodes (Patel et al., 2022; Kabakoff et al., 2024) with the prediction that stimulating the STS would eliminate the perceptual advantage of audiovisual speech, while stimulating the STG would interfere with auditory-only speech perception.

## References

[B1] Ahn E, Majumdar A, Lee TG, Brang D (2024) Evidence for a causal dissociation of the McGurk effect and congruent audiovisual speech perception via TMS to the left pSTS. Multisens Res 37:341–363. 10.1163/22134808-bja10129 39191410 PMC11388023

[B2] Aller M, Økland HS, MacGregor LJ, Blank H, Davis MH (2022) Differential auditory and visual phase-locking are observed during audio-visual benefit and silent lip-reading for speech perception. J Neurosci 42:6108–6120. 10.1523/JNEUROSCI.2476-21.2022 35760528 PMC9351641

[B3] Angelaki DE, Gu Y, DeAngelis GC (2009) Multisensory integration: psychophysics, neurophysiology, and computation. Curr Opin Neurobiol 19:452–458. 10.1016/j.conb.2009.06.008 19616425 PMC2749464

[B4] Arnal LH, Morillon B, Kell CA, Giraud A-L (2009) Dual neural routing of visual facilitation in speech processing. J Neurosci 29:13445–13453. 10.1523/JNEUROSCI.3194-09.2009 19864557 PMC6665008

[B5] Baum SH, Martin RC, Hamilton AC, Beauchamp MS (2012) Multisensory speech perception without the left superior temporal sulcus. Neuroimage 62:1825–1832. 10.1016/j.neuroimage.2012.05.034 22634292 PMC3408546

[B6] Beauchamp MS, Argall BD, Bodurka J, Duyn JH, Martin A (2004) Unraveling multisensory integration: patchy organization within human STS multisensory cortex. Nat Neurosci 7:1190–1192. 10.1038/nn1333 15475952

[B7] Beauchamp MS, Nath AR, Pasalar S (2010) fMRI-Guided transcranial magnetic stimulation reveals that the superior temporal sulcus is a cortical locus of the McGurk effect. J Neurosci 30:2414–2417. 10.1523/JNEUROSCI.4865-09.2010 20164324 PMC2844713

[B8] Chandrasekaran C, Trubanova A, Stillittano S, Caplier A, Ghazanfar AA (2009) The natural statistics of audiovisual speech. PLoS Comput Biol 5:e1000436. 10.1371/journal.pcbi.1000436 19609344 PMC2700967

[B9] Cohen MX (2014) Analyzing neural time series data: theory and practice, issues in clinical and cognitive neuropsychology. Cambridge, Massachusetts: The MIT Press.

[B10] Crosse MJ, Di Liberto GM, Lalor EC (2016) Eye can hear clearly now: inverse effectiveness in natural audiovisual speech processing relies on long-term crossmodal temporal integration. J Neurosci 36:9888–9895. 10.1523/JNEUROSCI.1396-16.2016 27656026 PMC6705572

[B11] Dahl CD, Logothetis NK, Kayser C (2009) Spatial organization of multisensory responses in temporal association cortex. J Neurosci 29:11924–11932. 10.1523/JNEUROSCI.3437-09.2009 19776278 PMC6666661

[B12] Davis MH, Johnsrude IS (2003) Hierarchical processing in spoken language comprehension. J Neurosci 23:3423–3431. 10.1523/JNEUROSCI.23-08-03423.2003 12716950 PMC6742313

[B13] Desikan RS, et al. (2006) An automated labeling system for subdividing the human cerebral cortex on MRI scans into gyral based regions of interest. Neuroimage 31:968–980. 10.1016/j.neuroimage.2006.01.021 16530430

[B14] Di Liberto GM, Crosse MJ, Lalor EC (2018) Cortical measures of phoneme-level speech encoding correlate with the perceived clarity of natural speech. eNeuro 5:ENEURO.0084-18.2018. 10.1523/ENEURO.0084-18.2018 PMC5900464 29662947

[B15] Dronkers NF, Wilkins DP, Van Valin RD, Redfern BB, Jaeger JJ (2004) Lesion analysis of the brain areas involved in language comprehension. Cognition 92:145–177. 10.1016/j.cognition.2003.11.002 15037129

[B16] Dubey A, Ray S (2019) Cortical electrocorticogram (ECoG) is a local signal. J Neurosci 39:4299–4311. 10.1523/JNEUROSCI.2917-18.2019 30914446 PMC6538865

[B17] Fischl B, Sereno MI, Dale AM (1999) Cortical surface-based analysis. II: Inflation, flattening, and a surface-based coordinate system. Neuroimage 9:195–207. 10.1006/nimg.1998.0396 9931269

[B18] Gau R, Bazin PL, Trampel R, Turner R, Noppeney U (2020) Resolving multisensory and attentional influences across cortical depth in sensory cortices. eLife 9:e46856. 10.7554/eLife.46856 31913119 PMC6984812

[B19] Gelman A, Stern H (2006) The difference between “significant” and “not significant” is not itself statistically significant. Am Stat 60:328–331. 10.1198/000313006X152649

[B20] Giraud A-L, Poeppel D (2012) Cortical oscillations and speech processing: emerging computational principles and operations. Nat Neurosci 15:511–517. 10.1038/nn.3063 22426255 PMC4461038

[B21] Hamilton LS, Edwards E, Chang EF (2018) A spatial map of onset and sustained responses to speech in the human superior temporal gyrus. Curr Biol 28:1860–1871.e4. 10.1016/j.cub.2018.04.033 29861132

[B22] Hamilton LS, Oganian Y, Hall J, Chang EF (2021) Parallel and distributed encoding of speech across human auditory cortex. Cell 184:4626–4639.e13. 10.1016/j.cell.2021.07.019 34411517 PMC8456481

[B23] Hickok G, Poeppel D (2004) Dorsal and ventral streams: a framework for understanding aspects of the functional anatomy of language. Cognition 92:67–99. 10.1016/j.cognition.2003.10.011 15037127

[B24] Hyafil A, Fontolan L, Kabdebon C, Gutkin B, Giraud A-L (2015) Speech encoding by coupled cortical theta and gamma oscillations. eLife 4:e06213. 10.7554/eLife.06213 26023831 PMC4480273

[B25] Kabakoff H, Yu L, Friedman D, Dugan P, Doyle WK, Devinsky O, Flinker A (2024) Timing and location of speech errors induced by direct cortical stimulation. Brain Commun 6:fcae053. 10.1093/braincomms/fcae053 38505231 PMC10948744

[B26] Karas PJ, Magnotti JF, Metzger BA, Zhu LL, Smith KB, Yoshor D, Beauchamp MS (2019) The visual speech head start improves perception and reduces superior temporal cortex responses to auditory speech. eLife 8:1–19. 10.7554/eLife.48116 PMC6687434 31393261

[B27] Karthik G, et al. (2021) Visual speech differentially modulates beta, theta, and high gamma bands in auditory cortex. Eur J Neurosci 54:7301–7317. 10.1111/ejn.15482 34587350 PMC8630510

[B28] Karthik G, Cao CZ, Demidenko MI, Jahn A, Stacey WC, Wasade VS, Brang D (2024) Auditory cortex encodes lipreading information through spatially distributed activity. Curr Biol 34:4021–4032.e5. 10.1016/j.cub.2024.07.073 39153482 PMC11387126

[B29] Kriegeskorte N, Simmons WK, Bellgowan PSF, Baker CI (2009) Circular analysis in systems neuroscience: the dangers of double dipping. Nat Neurosci 12:535–540. 10.1038/nn.2303 19396166 PMC2841687

[B30] Leske S, Dalal SS (2019) Reducing power line noise in EEG and MEG data via spectrum interpolation. Neuroimage 189:763–776. 10.1016/j.neuroimage.2019.01.026 30639330 PMC6456018

[B31] Magnotti JF, Wang Z, Beauchamp MS (2020) RAVE: comprehensive open-source software for reproducible analysis and visualization of intracranial EEG data. Neuroimage 223:117341. 10.1016/j.neuroimage.2020.117341 32920161 PMC7821728

[B32] Mégevand P, Mercier MR, Groppe DM, Zion Golumbic E, Mesgarani N, Beauchamp MS, Schroeder CE, Mehta AD (2020) Crossmodal phase reset and evoked responses provide complementary mechanisms for the influence of visual speech in auditory cortex. J Neurosci 40:8530–8542. 10.1523/JNEUROSCI.0555-20.2020 33023923 PMC7605423

[B33] Metzger BA, Magnotti JF, Wang Z, Nesbitt E, Karas PJ, Yoshor D, Beauchamp MS (2020) Responses to visual speech in human posterior superior temporal gyrus examined with iEEG deconvolution. J Neurosci 40:6938–6948. 10.1523/JNEUROSCI.0279-20.2020 32727820 PMC7470920

[B34] Michail G, Senkowski D, Holtkamp M, Wächter B, Keil J (2022) Early beta oscillations in multisensory association areas underlie crossmodal performance enhancement. Neuroimage 257:119307. 10.1016/j.neuroimage.2022.119307 35577024

[B35] Norman-Haignere SV, et al. (2022) Multiscale temporal integration organizes hierarchical computation in human auditory cortex. Nat Hum Behav 6:455–469. 10.1038/s41562-021-01261-y 35145280 PMC8957490

[B36] Nourski KV, Steinschneider M, Rhone AE, Kovach CK, Banks MI, Krause BM, Kawasaki H, Howard MA (2021) Electrophysiology of the human superior temporal sulcus during speech processing. Cereb Cortex 31:1131–1148. 10.1093/cercor/bhaa281 33063098 PMC7786351

[B37] Nourski KV, Howard MA (2015) Invasive recordings in the human auditory cortex. Handb Clin Neurol 129:225–244. 10.1016/B978-0-444-62630-1.00013-5 25726272

[B38] Ohki T, et al. (2016) Neural oscillations in the temporal pole for a temporally congruent audio-visual speech detection task. Sci Rep 6:37973. 10.1038/srep37973 27897244 PMC5126633

[B39] O’Sullivan AE, Crosse MJ, Liberto GMD, de Cheveigné A, Lalor EC (2021) Neurophysiological indices of audiovisual speech processing reveal a hierarchy of multisensory integration effects. J Neurosci 41:4991–5003. 10.1523/JNEUROSCI.0906-20.2021 33824190 PMC8197638

[B40] Ozker M, Schepers IM, Magnotti JF, Yoshor D, Beauchamp MS (2017) A double dissociation between anterior and posterior superior temporal gyrus for processing audiovisual speech demonstrated by electrocorticography. J Cogn Neurosci 29:1044–1060. 10.1162/jocn_a_01110 28253074 PMC5604231

[B41] Ozker M, Yoshor D, Beauchamp MS (2018) Converging evidence from electrocorticography and BOLD fMRI for a sharp functional boundary in superior temporal gyrus related to multisensory speech processing. Front Hum Neurosci 12:141. 10.3389/fnhum.2018.00141 29740294 PMC5928751

[B42] Patel P, Khalighinejad B, Herrero JL, Bickel S, Mehta AD, Mesgarani N (2022) Improved speech hearing in noise with invasive electrical brain stimulation. J Neurosci 42:3648–3658. 10.1523/JNEUROSCI.1468-21.2022 35347046 PMC9053855

[B44] Peelle JE, Davis MH (2012) Neural oscillations carry speech rhythm through to comprehension. Front Psychol 3:320. 10.3389/fpsyg.2012.00320 22973251 PMC3434440

[B45] Peelle JE, Sommers MS (2015) Prediction and constraint in audiovisual speech perception. Cortex 68:169–181. 10.1016/j.cortex.2015.03.006 25890390 PMC4475441

[B43] Peelle JE, Johnsrude IS, Davis MH (2010) Hierarchical processing for speech in human auditory cortex and beyond. Front Hum Neurosci 4:51. 10.3389/fnhum.2010.00051 20661456 PMC2907234

[B46] Poeppel D, Idsardi WJ, van Wassenhove V (2008) Speech perception at the interface of neurobiology and linguistics. Philos Trans R Soc Lond B Biol Sci 363:1071–1086. 10.1098/rstb.2007.2160 17890189 PMC2606797

[B47] Price CJ (2012) A review and synthesis of the first 20 years of PET and fMRI studies of heard speech, spoken language and reading. Neuroimage 62:816–847. 10.1016/j.neuroimage.2012.04.062 22584224 PMC3398395

[B48] Raij T, et al. (2010) Onset timing of cross-sensory activations and multisensory interactions in auditory and visual sensory cortices. Eur J Neurosci 31:1772–1782. 10.1111/j.1460-9568.2010.07213.x 20584181 PMC3008317

[B49] Rauschecker JP (1998) Parallel processing in the auditory cortex of primates. Audiol Neurootol 3:86–103. 10.1159/000013784 9575379

[B50] Ray S, Crone NE, Niebur E, Franaszczuk PJ, Hsiao SS (2008) Neural correlates of high-gamma oscillations (60–200 Hz) in macaque local field potentials and their potential implications in electrocorticography. J Neurosci 28:11526–11536. 10.1523/JNEUROSCI.2848-08.2008 18987189 PMC2715840

[B51] Rennig J, Beauchamp MS (2022) Intelligibility of audiovisual sentences drives multivoxel response patterns in human superior temporal cortex. Neuroimage 247:118796. 10.1016/j.neuroimage.2021.118796 34906712 PMC8819942

[B52] Ross LA, Saint-Amour D, Leavitt VM, Javitt DC, Foxe JJ (2007) Do you see what I am saying? Exploring visual enhancement of speech comprehension in noisy environments. Cereb Cortex 17:1147–1153. 10.1093/cercor/bhl024 16785256

[B53] Rowland BA, Quessy S, Stanford TR, Stein BE (2007) Multisensory integration shortens physiological response latencies. J Neurosci 27:5879–5884. 10.1523/JNEUROSCI.4986-06.2007 17537958 PMC6672269

[B54] Schepers IM, Yoshor D, Beauchamp MS (2015) Electrocorticography reveals enhanced visual cortex responses to visual speech. Cereb Cortex 25:4103–4110. 10.1093/cercor/bhu127 24904069 PMC4715246

[B55] Senkowski D, Schneider TR, Foxe JJ, Engel AK (2008) Crossmodal binding through neural coherence: implications for multisensory processing. Trends Neurosci 31:401–409. 10.1016/j.tins.2008.05.002 18602171

[B56] Shan T, Wenner CE, Xu C, Duan Z, Maddox RK (2022) Speech-in-noise comprehension is improved when viewing a deep-neural-network-generated talking face. Trends Hear 26:23312165221136934. 10.1177/23312165221136934 36384325 PMC9677167

[B58] Stein BE, Meredith MA (1993) The merging of the senses. Cambridge, MA: MIT Press.

[B59] Stein BE, Stanford TR (2008) Multisensory integration: current issues from the perspective of the single neuron. Nat Rev Neurosci 9:255–266. 10.1038/nrn2331 18354398

[B57] Stein BE, Stanford TR, Ramachandran R, Perrault TJ, Rowland BA (2009) Challenges in quantifying multisensory integration: alternative criteria, models, and inverse effectiveness. Exp Brain Res 198:113–126. 10.1007/s00221-009-1880-8 19551377 PMC3056521

[B60] Sumby WH, Pollack I (1954) Visual contribution to speech intelligibility in noise. J Acoust Soc Am 26:212–215. 10.1121/1.1907309

[B61] Thézé R, Gadiri MA, Albert L, Provost A, Giraud A-L, Mégevand P (2020a) Animated virtual characters to explore audio-visual speech in controlled and naturalistic environments. Sci Rep 10:15540. 10.1038/s41598-020-72375-y 32968127 PMC7511320

[B62] Thézé R, Giraud A-L, Mégevand P (2020b) The phase of cortical oscillations determines the perceptual fate of visual cues in naturalistic audiovisual speech. Sci Adv 6:eabc6348. 10.1126/sciadv.abc6348 33148648 PMC7673697

[B63] van Bree S, Sohoglu E, Davis MH, Zoefel B (2021) Sustained neural rhythms reveal endogenous oscillations supporting speech perception. PLoS Biol 19:e3001142. 10.1371/journal.pbio.3001142 33635855 PMC7946281

[B64] van Wassenhove V, Grant KW, Poeppel D (2005) Visual speech speeds up the neural processing of auditory speech. Proc Natl Acad Sci U S A 102:1181–1186. 10.1073/pnas.0408949102 15647358 PMC545853

[B65] Varano E, Vougioukas K, Ma P, Petridis S, Pantic M, Reichenbach T (2021) Speech-driven facial animations improve speech-in-noise comprehension of humans. Front Neurosci 15:781196. 10.3389/fnins.2021.781196 35069100 PMC8766421

[B66] Wallace MT, Wilkinson LK, Stein BE (1996) Representation and integration of multiple sensory inputs in primate superior colliculus. J Neurophysiol 76:1246–1266. 10.1152/jn.1996.76.2.1246 8871234

[B67] Wang Z, Magnotti JF, Zhang X, Beauchamp MS (2023) YAEL: Your Advanced Electrode Localizer. eNeuro 10:ENEURO.0328-23.2023. 10.1523/ENEURO.0328-23.2023 PMC10591275 37857509

[B68] Yi HG, Leonard MK, Chang EF (2019) The encoding of speech sounds in the superior temporal gyrus. Neuron 102:1096–1110. 10.1016/j.neuron.2019.04.023 31220442 PMC6602075

[B69] Yu Y, Lado A, Zhang Y, Magnotti JF, Beauchamp MS (2024) Synthetic faces generated with the facial action coding system or deep neural networks improve speech-in-noise perception, but not as much as real faces. Front Neurosci 18:1379988. 10.3389/fnins.2024.1379988 38784097 PMC11111898

[B70] Zhang Y, Rennig J, Magnotti JF, Beauchamp MS (2023) Multivariate fMRI responses in superior temporal cortex predict visual contributions to, and individual differences in, the intelligibility of noisy speech. Neuroimage 278:120271. 10.1016/j.neuroimage.2023.120271 37442310 PMC10460966

[B71] Zhu LL, Beauchamp MS (2017) Mouth and voice: a relationship between visual and auditory preference in the human superior temporal sulcus. J Neurosci 37:2697–2708. 10.1523/JNEUROSCI.2914-16.2017 28179553 PMC5354323

